# Supplementary material for: Empirical methods for controlling false positives and estimating confidence in ChIP-Seq peaks
Source: BMC Bioinformatics. 2008 Dec 5;9:523. doi: 10.1186/1471-2105-9-523 (PMC2628906; doi:10.1186/1471-2105-9-523)
Supplement: Additional File 1 — A variety of html documents from the USeq web site detailing the available applications, their best usage, output file type descriptions, command line menus, etc. [file 1471-2105-9-523-S1.zip › USeqUserGuides/JavaDoc.zip!/JavaDoc/gata/main/GATAUtil.html]

GATAUtil


|  |  |  |
| --- | --- | --- |
| **Overview** \| **Package** \| **Class** \| **Use** \| **Tree** \| **Deprecated** \| **Index** \| **Help** | |  |
| **PREV CLASS**   **NEXT CLASS** | **FRAMES**    **NO FRAMES**     **All Classes** |  |
| SUMMARY: NESTED \| FIELD \| CONSTR \| METHOD | DETAIL: FIELD \| CONSTR \| METHOD |  |


---


## gata.main Class GATAUtil

```
java.lang.Object
  gata.main.GATAUtil
```

---

``` public class GATAUtil extends java.lang.Object ```

**Author:**
:   Nix
    Master utility class for GATAPlotter. All static.

---

| **Field Summary** | |
| --- | --- |
| `static java.awt.Color[]` | `COLORS`             Color array for tracks, yup I'm lazy |


**Constructor Summary**

`GATAUtil()`


| **Method Summary** | |
| --- | --- |
| `static java.awt.geom.Line2D.Double[]` | `ALToLine2DArray(java.util.ArrayList al)` |
| `static java.lang.String` | `ALToString(java.util.ArrayList al)` |
| `static int[]` | `calculateEffectiveMandN(double m, double n, double K, double H)`             Calculates effective m and n using BLAST parameters, returning int[m', n'] |
| `static java.lang.String` | `calculateEValue(double lambdaNats, double K, int rawS, double effectiveN, double effectiveM)` |
| `static boolean` | `checkFile(java.lang.String par)` |
| `static double` | `convertBitScoreToRaw(double lambdaNats, double K, double bitS)` |
| `static int` | `convertPlusToNumOrientation(java.lang.String orientation)` |
| `static double` | `convertRawScoreToBit(double lambdaNats, double K, double rawS)`             Converts a raw S score to a bit score given lambda (in nats) and K |
| `static void` | `deleteFile(java.lang.String fullPathFileName)` |
| `static int` | `DNAStat(java.lang.String seq1, java.lang.String seq2, int match, int misMatch, int gapCreate, int gapExtend)` |
| `static void` | `drawLabels(java.util.ArrayList labels, java.awt.Font font, java.awt.Graphics2D g2)` |
| `static void` | `drawLines(java.awt.geom.Line2D.Double[] lines, java.awt.Graphics2D g2)`             draws Shapes |
| `static java.util.ArrayList` | `extractStartEnds(ExonIntron[] exons)`             Deep copies start stops from ExonIntron objects to an ArrayList |
| `static int[][]` | `extractStartEndsInts(ExonIntron[] exons)` |
| `static java.util.ArrayList` | `fetchArrayList(java.io.File file)` |
| `static double[]` | `fetchBLASTParams(int match, int misMatch)`             Returns Lambda and K or null for a given match and misMatch from BLASTN. |
| `static java.util.ArrayList` | `fetchIntrons(java.util.ArrayList exons)`             Takes an ArrayList of exon startStop int[2]'s, returns an ArrayList of int[2]'s representing the start,stop for each intron |
| `static java.lang.Object` | `fetchObject(java.io.File file)` |
| `static java.awt.RenderingHints` | `fetchRenderingHints()`             Map of hints for high quality/ slow rendering |
| `static int` | `findHighestInt(int[] ints)` |
| `static java.lang.String` | `genDashes(java.lang.String seq1, java.lang.String seq2)` |
| `static int[]` | `getBaseNumArray(java.lang.String s, int index)` |
| `static boolean` | `isEmpty(java.lang.String text)`             Method to check if a string is null or empty after trimming |
| `static boolean` | `isStringEmpty(java.lang.String string)` |
| `static java.util.ArrayList` | `joinSegments(java.util.ArrayList segs)`             Takes an ArrayList of int[2]'s representing start stop coords kills the dups, and those cotained within another seg, will merge overlapping segments. |
| `static java.util.ArrayList` | `makeArrowAndButt(int start, int end, int upOrDown, double yCoord, int orientation, double pixPerNt, double ntAtX, double height)`             Makes an arrow and butt but no base line. |
| `static java.util.ArrayList` | `makeArrowLine(int start, int end, int upOrDown, double yCoord, int orientation, double pixPerNt, double ntAtX, double height)`             Makes an arrow line with half arrow up for -1 or down for 1. |
| `static java.awt.geom.GeneralPath` | `makeArrowLine(int start, int end, int upOrDown, float yCoord, int orientation, float pixPerNt, float ntAtX, float heightArrowButt, float lineThickness)`             Makes an arrow line with half arrow up for -1 or down for 1. |
| `static javax.swing.JButton` | `makeButton(java.lang.String text, int size, java.awt.event.ActionListener action, javax.swing.JPanel panel)` |
| `static double` | `makeDoubleInputDialog(java.lang.String title, java.lang.String comment, double suggestion)` |
| `static double` | `makeDoublePercentInputDialog(java.lang.String title, java.lang.String comment, double suggestion)` |
| `static javax.swing.JTextField` | `makeField(javax.swing.JPanel panel, java.lang.String text, javax.swing.JFrame frame, javax.swing.JFileChooser chooser, FileFocusListener focus)` |
| `static javax.swing.JTextField` | `makeFieldSimple(javax.swing.JPanel panel, java.lang.String text)` |
| `static int` | `makeIntInputDialog(java.lang.String title, java.lang.String comment, int suggestion)` |
| `static javax.swing.JLabel` | `makeLabel(javax.swing.JPanel panel, java.lang.String text, int size, int thickness)` |
| `static java.util.ArrayList` | `makeLabel(java.lang.String label, int start, int end, double pixPerNt, double ntAtX, double yCoor)` |
| `static java.awt.geom.Line2D.Double` | `makeLine(int start, int end, double yCoord, double pixPerNt, double ntAtX)`             Converts an int[2] (startStop) in nucleotides to a Line2D.Double |
| `static java.awt.geom.Rectangle2D.Double` | `makeRectangle(int start, int end, double startY, double stopY, double pixPerNt, double ntAtX)`             Returns a rectangle given start stop in nucleotides and y coords in pixels |
| `static java.util.ArrayList` | `makeSegments(java.util.ArrayList segs, double yCoord, double pixPerNt, double ntAtX)`             Converts an ArrayList of int[2]'s (startStop) in nucleotides to an ArrayList of Line2D.Double's |
| `static java.util.ArrayList` | `makeSegmentsInt(int[][] segs, double yCoord, double pixPerNt, double ntAtX)`             Converts an int[x][2]'s (startStop) in nucleotides to an ArrayList of Line2D.Double's |
| `static Alignment[]` | `makeSubAligns(LocalAlignment[] locs)` |
| `static LocalAlignment[]` | `mergeLAArrays(LocalAlignment[] a, LocalAlignment[] b)` |
| `static java.util.ArrayList` | `objectArraytoAL(java.lang.Object[] objs)`             Converts an Object[] to an ArrayList |
| `static int[]` | `optimizeAlignmentScore(java.lang.String seq1, java.lang.String seq2, int match, int misMatch, int gapCreate, int gapExtend, int scoreCutOff)` |
| `static java.lang.String` | `parseDNAFileName(java.lang.String s)` |
| `static void` | `saveArrayList(java.io.File file, java.util.ArrayList al)` |
| `static void` | `saveObject(java.io.File file, java.lang.Object ob)` |
| `static void` | `setBooleanArray(boolean[] array, boolean value)`             Takes an array of boolean[] and sets all to the boolean value |
| `static int[]` | `setButton(javax.swing.JButton but, int x, int y)` |
| `static int[]` | `setChooser(javax.swing.JFileChooser choos, int x, int y, int width)` |
| `static int[]` | `setField(javax.swing.JTextField field, int x, int y, int width)` |
| `static void` | `setFloatArray(float[] array, float value)`             Takes an array of float[] and sets all to the float value |
| `static int[]` | `setLabel(javax.swing.JLabel lab, int x, int y)` |
| `static void` | `setScrollPaneScrollBarIncrement(javax.swing.JScrollPane scrollPane, int increment)`             Sets the scroll bar move increments, vert and horiz, in a scroll pane |
| `static void` | `showFileChooserDialogBox(javax.swing.JTextField fieldRef, javax.swing.JFrame frame, javax.swing.JFileChooser chooser)` |
| `static java.util.ArrayList` | `sortSegments(java.util.ArrayList segs)`             Sorts an ArrayList of int[2]'s representing start, end |
| `static java.lang.String` | `spaces(java.lang.String seq, int number)` |
| `static void` | `throwWarning(javax.swing.JFrame frame, javax.swing.JTextField field, java.lang.String text)` |
| `static void` | `writeString(java.lang.String data, java.lang.String fullPathFileName)` |

**Methods inherited from class java.lang.Object**

`equals, getClass, hashCode, notify, notifyAll, toString, wait, wait, wait`

**Field Detail**

### COLORS

```
public static final java.awt.Color[] COLORS
```

:   Color array for tracks, yup I'm lazy


**Constructor Detail**

### GATAUtil

```
public GATAUtil()
```


**Method Detail**

### setBooleanArray

```
public static void setBooleanArray(boolean[] array,
                                   boolean value)
```

:   Takes an array of boolean[] and sets all to the boolean value

---


### setFloatArray

```
public static void setFloatArray(float[] array,
                                 float value)
```

:   Takes an array of float[] and sets all to the float value

---


### setScrollPaneScrollBarIncrement

```
public static void setScrollPaneScrollBarIncrement(javax.swing.JScrollPane scrollPane,
                                                   int increment)
```

:   Sets the scroll bar move increments, vert and horiz, in a scroll pane

---


### makeDoublePercentInputDialog

```
public static double makeDoublePercentInputDialog(java.lang.String title,
                                                  java.lang.String comment,
                                                  double suggestion)
```

---


### makeDoubleInputDialog

```
public static double makeDoubleInputDialog(java.lang.String title,
                                           java.lang.String comment,
                                           double suggestion)
```

---


### makeIntInputDialog

```
public static int makeIntInputDialog(java.lang.String title,
                                     java.lang.String comment,
                                     int suggestion)
```

---


### throwWarning

```
public static void throwWarning(javax.swing.JFrame frame,
                                javax.swing.JTextField field,
                                java.lang.String text)
```

---


### setButton

```
public static int[] setButton(javax.swing.JButton but,
                              int x,
                              int y)
```

---


### makeButton

```
public static javax.swing.JButton makeButton(java.lang.String text,
                                             int size,
                                             java.awt.event.ActionListener action,
                                             javax.swing.JPanel panel)
```

---


### showFileChooserDialogBox

```
public static void showFileChooserDialogBox(javax.swing.JTextField fieldRef,
                                            javax.swing.JFrame frame,
                                            javax.swing.JFileChooser chooser)
```

---


### checkFile

```
public static boolean checkFile(java.lang.String par)
```

---


### makeField

```
public static javax.swing.JTextField makeField(javax.swing.JPanel panel,
                                               java.lang.String text,
                                               javax.swing.JFrame frame,
                                               javax.swing.JFileChooser chooser,
                                               FileFocusListener focus)
```

---


### makeFieldSimple

```
public static javax.swing.JTextField makeFieldSimple(javax.swing.JPanel panel,
                                                     java.lang.String text)
```

---


### makeLabel

```
public static javax.swing.JLabel makeLabel(javax.swing.JPanel panel,
                                           java.lang.String text,
                                           int size,
                                           int thickness)
```

---


### setChooser

```
public static int[] setChooser(javax.swing.JFileChooser choos,
                               int x,
                               int y,
                               int width)
```

---


### setField

```
public static int[] setField(javax.swing.JTextField field,
                             int x,
                             int y,
                             int width)
```

---


### setLabel

```
public static int[] setLabel(javax.swing.JLabel lab,
                             int x,
                             int y)
```

---


### deleteFile

```
public static void deleteFile(java.lang.String fullPathFileName)
```

---


### convertRawScoreToBit

```
public static double convertRawScoreToBit(double lambdaNats,
                                          double K,
                                          double rawS)
```

:   Converts a raw S score to a bit score given lambda (in nats) and K

---


### convertBitScoreToRaw

```
public static double convertBitScoreToRaw(double lambdaNats,
                                          double K,
                                          double bitS)
```

---


### calculateEValue

```
public static java.lang.String calculateEValue(double lambdaNats,
                                               double K,
                                               int rawS,
                                               double effectiveN,
                                               double effectiveM)
```

---


### calculateEffectiveMandN

```
public static int[] calculateEffectiveMandN(double m,
                                            double n,
                                            double K,
                                            double H)
```

:   Calculates effective m and n using BLAST parameters, returning int[m', n']

---


### DNAStat

```
public static int DNAStat(java.lang.String seq1,
                          java.lang.String seq2,
                          int match,
                          int misMatch,
                          int gapCreate,
                          int gapExtend)
```

---


### findHighestInt

```
public static int findHighestInt(int[] ints)
```

---


### genDashes

```
public static java.lang.String genDashes(java.lang.String seq1,
                                         java.lang.String seq2)
```

---


### getBaseNumArray

```
public static int[] getBaseNumArray(java.lang.String s,
                                    int index)
```

---


### makeSubAligns

```
public static Alignment[] makeSubAligns(LocalAlignment[] locs)
```

---


### mergeLAArrays

```
public static LocalAlignment[] mergeLAArrays(LocalAlignment[] a,
                                             LocalAlignment[] b)
```

---


### optimizeAlignmentScore

```
public static int[] optimizeAlignmentScore(java.lang.String seq1,
                                           java.lang.String seq2,
                                           int match,
                                           int misMatch,
                                           int gapCreate,
                                           int gapExtend,
                                           int scoreCutOff)
```

---


### parseDNAFileName

```
public static java.lang.String parseDNAFileName(java.lang.String s)
```

---


### spaces

```
public static java.lang.String spaces(java.lang.String seq,
                                      int number)
```

---


### writeString

```
public static void writeString(java.lang.String data,
                               java.lang.String fullPathFileName)
```

---


### fetchRenderingHints

```
public static java.awt.RenderingHints fetchRenderingHints()
```

:   Map of hints for high quality/ slow rendering

---


### drawLabels

```
public static void drawLabels(java.util.ArrayList labels,
                              java.awt.Font font,
                              java.awt.Graphics2D g2)
```

---


### makeLabel

```
public static java.util.ArrayList makeLabel(java.lang.String label,
                                            int start,
                                            int end,
                                            double pixPerNt,
                                            double ntAtX,
                                            double yCoor)
```

---


### ALToLine2DArray

```
public static java.awt.geom.Line2D.Double[] ALToLine2DArray(java.util.ArrayList al)
```

---


### drawLines

```
public static void drawLines(java.awt.geom.Line2D.Double[] lines,
                             java.awt.Graphics2D g2)
```

:   draws Shapes

---


### makeSegments

```
public static java.util.ArrayList makeSegments(java.util.ArrayList segs,
                                               double yCoord,
                                               double pixPerNt,
                                               double ntAtX)
```

:   Converts an ArrayList of int[2]'s (startStop) in nucleotides to an ArrayList of Line2D.Double's

---


### makeSegmentsInt

```
public static java.util.ArrayList makeSegmentsInt(int[][] segs,
                                                  double yCoord,
                                                  double pixPerNt,
                                                  double ntAtX)
```

:   Converts an int[x][2]'s (startStop) in nucleotides to an ArrayList of Line2D.Double's

---


### makeLine

```
public static java.awt.geom.Line2D.Double makeLine(int start,
                                                   int end,
                                                   double yCoord,
                                                   double pixPerNt,
                                                   double ntAtX)
```

:   Converts an int[2] (startStop) in nucleotides to a Line2D.Double

---


### makeRectangle

```
public static java.awt.geom.Rectangle2D.Double makeRectangle(int start,
                                                             int end,
                                                             double startY,
                                                             double stopY,
                                                             double pixPerNt,
                                                             double ntAtX)
```

:   Returns a rectangle given start stop in nucleotides and y coords in pixels

---


### makeArrowAndButt

```
public static java.util.ArrayList makeArrowAndButt(int start,
                                                   int end,
                                                   int upOrDown,
                                                   double yCoord,
                                                   int orientation,
                                                   double pixPerNt,
                                                   double ntAtX,
                                                   double height)
```

:   Makes an arrow and butt but no base line. Half arrow/butt up for -1 or down for 1. Start
    and end are really just 5 ' and 3' coordinates, start should always be less
    than end. Orientation is 1 for + (arrow pointing right), -1 for - (left).
    yCoord is for the y-coordinate to draw the main line. Returns an
    ArrayList of Line2D.doubles.

---


### makeArrowLine

```
public static java.util.ArrayList makeArrowLine(int start,
                                                int end,
                                                int upOrDown,
                                                double yCoord,
                                                int orientation,
                                                double pixPerNt,
                                                double ntAtX,
                                                double height)
```

:   Makes an arrow line with half arrow up for -1 or down for 1. Start
    and end are really just 5 ' and 3' coordinates, start should always be less
    than end. Orientation is 1 for + (arrow pointing right), -1 for - (left).
    yCoord is for the y-coordinate to draw the main line. Returns an
    ArrayList of Line2D.doubles.

---


### makeArrowLine

```
public static java.awt.geom.GeneralPath makeArrowLine(int start,
                                                      int end,
                                                      int upOrDown,
                                                      float yCoord,
                                                      int orientation,
                                                      float pixPerNt,
                                                      float ntAtX,
                                                      float heightArrowButt,
                                                      float lineThickness)
```

:   Makes an arrow line with half arrow up for -1 or down for 1. Start
    and end are really just 5 ' and 3' coordinates, start should always be less
    than end. Orientation is 1 for + (arrow pointing right), -1 for - (left).
    yCoord is for the y-coordinate to draw the main line. Line thickness is
    needed to calculate whether to draw an arrow or not, for short segments.
    Will always return a line of at least 1 pixel.

---


### fetchBLASTParams

```
public static double[] fetchBLASTParams(int match,
                                        int misMatch)
```

:   Returns Lambda and K or null for a given match and misMatch from BLASTN.
    Some combinations of match/misMatch are not computed by BLASTN!

---


### saveArrayList

```
public static void saveArrayList(java.io.File file,
                                 java.util.ArrayList al)
```

---


### saveObject

```
public static void saveObject(java.io.File file,
                              java.lang.Object ob)
```

---


### isStringEmpty

```
public static boolean isStringEmpty(java.lang.String string)
```

---


### fetchArrayList

```
public static java.util.ArrayList fetchArrayList(java.io.File file)
```

---


### fetchObject

```
public static java.lang.Object fetchObject(java.io.File file)
```

---


### isEmpty

```
public static boolean isEmpty(java.lang.String text)
```

:   Method to check if a string is null or empty after trimming

---


### objectArraytoAL

```
public static java.util.ArrayList objectArraytoAL(java.lang.Object[] objs)
```

:   Converts an Object[] to an ArrayList

---


### extractStartEnds

```
public static java.util.ArrayList extractStartEnds(ExonIntron[] exons)
```

:   Deep copies start stops from ExonIntron objects to an ArrayList

---


### extractStartEndsInts

```
public static int[][] extractStartEndsInts(ExonIntron[] exons)
```

---


### convertPlusToNumOrientation

```
public static int convertPlusToNumOrientation(java.lang.String orientation)
```

---


### fetchIntrons

```
public static java.util.ArrayList fetchIntrons(java.util.ArrayList exons)
```

:   Takes an ArrayList of exon startStop int[2]'s, returns an ArrayList of int[2]'s representing
    the start,stop for each intron

---


### ALToString

```
public static java.lang.String ALToString(java.util.ArrayList al)
```

---


### sortSegments

```
public static java.util.ArrayList sortSegments(java.util.ArrayList segs)
```

:   Sorts an ArrayList of int[2]'s representing start, end

---


### joinSegments

```
public static java.util.ArrayList joinSegments(java.util.ArrayList segs)
```

:   Takes an ArrayList of int[2]'s representing start stop coords
    kills the dups, and those cotained within another seg, will
    merge overlapping segments.


---


|  |  |  |
| --- | --- | --- |
| **Overview** \| **Package** \| **Class** \| **Use** \| **Tree** \| **Deprecated** \| **Index** \| **Help** | |  |
| **PREV CLASS**   **NEXT CLASS** | **FRAMES**    **NO FRAMES**     **All Classes** |  |
| SUMMARY: NESTED \| FIELD \| CONSTR \| METHOD | DETAIL: FIELD \| CONSTR \| METHOD |  |


---
